# Multi‐omics analysis reveals the interaction between the complement system and the coagulation cascade in the development of endometriosis

**DOI:** 10.1038/s41598-021-90112-x

**Published:** 2021-06-07

**Authors:** Liang Yu, Huaji Shen, Xiaohan Ren, Anqi Wang, Shu Zhu, Yafeng Zheng, Xiuli Wang

**Affiliations:** 1 The State Key Lab of Reproductive, Department of Obstetrics and Gynecology, Jiangsu Province Hospital, Nanjing, 210029 China; 2 Department of Obstetrics and Gynecology, the Affiliated Changzhou No. 2 People’s Hospital of Nanjing Medical University, Changzhou, 213000 China

**Keywords:** Biomarkers, Medical research, Molecular medicine, Diseases

## Abstract

Endometriosis (EMS) is a disease that shows immune dysfunction and chronic inflammation characteristics, suggesting a role of complement system in its pathophysiology. To find out the hub genes and pathways involved in the pathogenesis of EMs, three raw microarray datasets were recruited from the Gene Expression Omnibus database (GEO). Then, a series of bioinformatics technologies including gene ontology (GO), Hallmark pathway enrichment, protein–protein interaction (PPI) network and gene co-expression correlation analysis were performed to identify hub genes. The hub genes were further verified by the Real-time quantitative polymerase chain reaction (RT-PCR) and Western Blot (WB). We identified 129 differentially expressed genes (DEGs) in EMs, of which 78 were up-regulated and 51 were down-regulated. Through GO functional enrichment analysis, we found that the DEGs are mainly enriched in cell adhesion, extracellular matrix remodeling, chemokine regulation, angiogenesis regulation, epithelial cell proliferation, et al. In Hallmark pathway enrichment analysis, coagulation pathway showed great significance and the terms in which included the central complement factors. Moreover, the genes were dominating in PPI network. Combined co-expression analysis with experimental verification, we found that the up-regulated expression of complement (C1S, C1QA, C1R, and C3) was positively related to tissue factor (TF) in EMs. In this study, we discovered the over expression complement and the positive correlation between complement and TF in EMs, which suggested that interaction of complement and coagulation system may play a role within the pathophysiology of EMS.

## Introduction

Endometriosis (EMS), characterized by the presence of functional endometrial tissue outside the uterine cavity, is a common disease affecting up to 10–15% of reproductive-age women and is closely related to dysmenorrhea, chronic pelvic pain and infertility^1^. Among the hypotheses that have been proposed to explain the pathology of EMS, Sampson’s theory of retrograde menstruation is the most widely accepted etiology^2^. “Immunosurveillance” dysfunction was a critical facilitator of ectopic endometrial tissue implantation and growth^3^. EMS was considered as a chronic inflammatory disease^4^. One of the important immune mechanisms involved in the peritoneal clearance, inflammation and autoimmune in EMS was the complement system^5–7^. The close association of coagulation and inflammatory conditions has been known for a long time^8^. Current studies have showed that women with EMS appeared to be in a state of hypercoagulability and this coagulation dysfunction potentially attributed to the inflammatory nature of the ectopic lesions^9–11^. In recent years, the massive data of gene expression profile generated from high-throughput sequencing technology make it possible for us to explore the intrinsic mechanism of diseases at the molecular level^12^. Some meaningful researches have been conducted to identify genes involved in the development of EMS^13,14^. In this study, we integrated data sets from multiple studies and performed deep bioinformatic analyses to identify differentially expressed genes (DEGs) and important pathways participating in the development of EMS. Through pathway enrichment analysis, the coagulation process containing the central complement factors was indicated as the significant component of EMS. Real-time quantitative polymerase chain reaction (RT-PCR) and Western Blot (WB) were adopted to validate these results in women with or without EMS. After evaluating and comparing the expression of hub genes in EMS and control women, we found that the overexpressed state of complement was positively related to tissue factor (TF) in EMS. These suggested the existence of crosstalk between complement and coagulation in EMS.

## Method

### Data acquistion and differentially expressed genes (DEGs) identification

Three datasets (GSE23339, GSE7305, and GSE25628) were selected for our analysis. In detail, the gene set GSE23339 was annotated by GPL6102 platform, which included 10 endometriosis tissue samples and 9 normal endometrial tissue samples (as of control samples); GSE7305 was annotated by GPL570 platform, the data in which consist of 10 endometriosis tissue samples and 10 normal endometrial tissue samples; GSE25628 was annotated by GPL571 platform, which included 16 endometriosis and 6 normal endometrial samples. The platform annotation and series matrix files were downloaded for follow-up analyses.

Next, the orignial file obtained from GEO databases were preprocessed in R software. Samples of each dataset were normalized by the Robust Multi-array Average (RMA) function in the Affy package^15^. And then we used SVA package to eliminate the inter-batch difference between groups^16^. Multiple probes relating to the same gene were summarized as the median value for further analysis^17^. In our analysis, DEGs were defined with a threshold of p value < 0.05 and |log (FC)|> 1. DEGs in each database were displayed with the volcano map. Overlapping DEGs within three databases were shown with Venn diagrams.

### Protein–protein interaction (PPI) network analysis

The PPI of DEGs-encoded proteins was confirmed by STRING^18^ (version 11.0), with search limitation to Homo sapiens and a score > 0.400 correspondingsto high confidence and statistical significance. After that, Cytoscape software (version 3.7.1) was used to construct the network^19^. Moreover, the cytohubba plug-in unit of Cytoscape software was used for the identification of the top 20 hub genes according to the count of interactions between genes.

### Gene ontology and pathway enrichment analysis

Gene ontology (GO) Biological Processes of DEGs were analyzed using "clusterProfiler" package in R software^20^. P-value < 0.05 was defined as the cutoff value. GO considers annotates gene function from three aspects: molecular function(MF), cellular component(CC) and biological process(BP)^21^. Among these, Molecular functions define molecular processes, Cellular components define locations where molecular processes occur and Biological processes define biological programs comprised of regulated factors. To remove repetition and ensure the authenticity of the results, we merged the terms using simplify function in the GOplot package. The Hallmark pathway enrichment analysis was performed in Metascape16 (https://metascape.org/gp/index.html#/main/step3)(22). P-value < 0.05 was defined as the cutoff value.

### Gene co-expression correlation analysis

To identify the correlation between hub genes, GSE141549 was downloaded as a validation set. GSE141549 was annotated by GPL10558 platform, the data in which consist of 198 endometriosis samples and 147 normal samples. We used R to build a co-expression network and visualize it.

### Clinical sample collection

Eutopic endometrial tissues and control endometrium were obtained respectively from three patients with ovarian endometriosis (OE) and three women with benign ovarian teratoma, which were diagnosed by pathology after surgery. According to the American Fertility Society revised (AFS-r), these 3 EMs cases were all stage IV. All participants have written informed consent under the study protocol approved by the Ethics Committee of Changzhou Second People's Hospital (Changzhou, China). All of the endometrial tissues were collected at the proliferative phase during the menstrual cycle. None of the recruited women smoked or had taken any anticoagulant drugs or steroid hormones 3 months prior to the operation. There was no significant difference in age between the two groups (P > 0.05).

### Quantitative-Polymerase chain reaction (qPCR)

Firstly, we isolated total RNA by using Trizol (Invitrogen) according to the manufacturer's protocol. The PrimeScript RT Master Mix (Takara, JPN) was used for the synthesis of cDNA. Quantitative-Polymerase chain reaction was performed using the SYBR Green assay to evaluate the expression of hub genes on the basis of manufacturer's instructions (Applied Biosystems, USA). The typical qPCR cycle were as followed: Pre‑denaturation at 95˚C for 30 s, followed by 40 cycles of denaturation at 95˚C for 5 s, annealing at 60˚C for 10 s and extension at 70–72˚C for 30 s^23^. We used GAPDH to normalize the expression of hub genes. And then, The relative expression level of the target amplicon was calculated between the EMs group and the control group. Hub genes and GAPDH primers were designed and synthesized by Sangon Biotech Co., Ltd. (Shanghai, China). The primers’ sequences were showed in Table 1.

**Table 1:** Detail information of primers.

Primer name	Primer forward sequences (5’-3’)	Primer reverse sequences (5’-3’)
C1R	TTCCCCAAGCCTTACCCCAA	GCTGGAAGACGAGCTTCACC
C1S	ACTGTGCGTATGACTCAGTGC	GGGGATTGTTACTGCTCCTCT
C1QA	TCTGCACTGTACCCGGCTA	CCCTGGTAAATGTGACCCTTTT
C3	CGCAACAAGTTCGTGACCG	GATGCCTTCCGGGTTCTCAAT
P2RY14	AATCTAGCCGCAACATATTCAGC	GTCTGACTCTTTGTGTAGGGGAT
SEPRING1	CTGGCTGGGGATAGAGCCT	GAGATAACTGTTGTTGCGACCT
TF	GAGATAACTGTTGTTGCGACCT	CATCGGATGGAATGACGCTTT

### Proteins extraction and Western Blot (WB)

Total proteins were extracted from human EMs tissue with Western and IP lysis buffer (Beyotime, P0013; Beijing). Protein concentration was measured using the BCA reagent kit (Pierce, 23,227). The proteins were resolved by 8%-12% SDS-PAGE and then blotted onto polyvinylidene fluoride (PVDF) membranes. Membranes were blocked in TBS/0.1% Tween-20 (TBST) containing 5% skimmed milk powder for 1 h at room temperature. Hub proteins and GAPDH antibodies were diluted with 1:300 (AtaGenix, Wuhan) and 1:2000 (AtaGenix, Wuhan) before incubation with 2 h at room temperature. The secondary antibody [anti-rabbit IgG (H + L) biotinylated antibody (CST, USA)] was incubated with 2 h at room temperature^24^.

### Statistical analysis

All data statistics are analyzed using R software. P value < 0.05 is considered statistically significant. Independent t-test was used to compare the continuous variables of normal distribution and Mann Whitney U-test was used to compare the continuous variables of skew distribution.

### Ethics approval, informed consent and experimental approval

This study was approved by the Ethics Committee of the Affiliated Changzhou No.2 People’s Hospital of Nanjing Medical University, China. All patients have signed the informed consent for the study protocol and reserve the right to withdraw at any time. The experimental scheme was approved by the academic committee of Ethics Committee of the Affiliated Changzhou No.2 People’s Hospital of Nanjing Medical University, and the experimental methods were carried out in accordance with the guidelines of the academic committee. The study was conducted according to common standard guidelines for clinical trials (Declaration of Helsinki).

## Results

### Identification of differentially expressed genes (DEGs) in EMS using integrated bioinformatics

**Figure 1:**
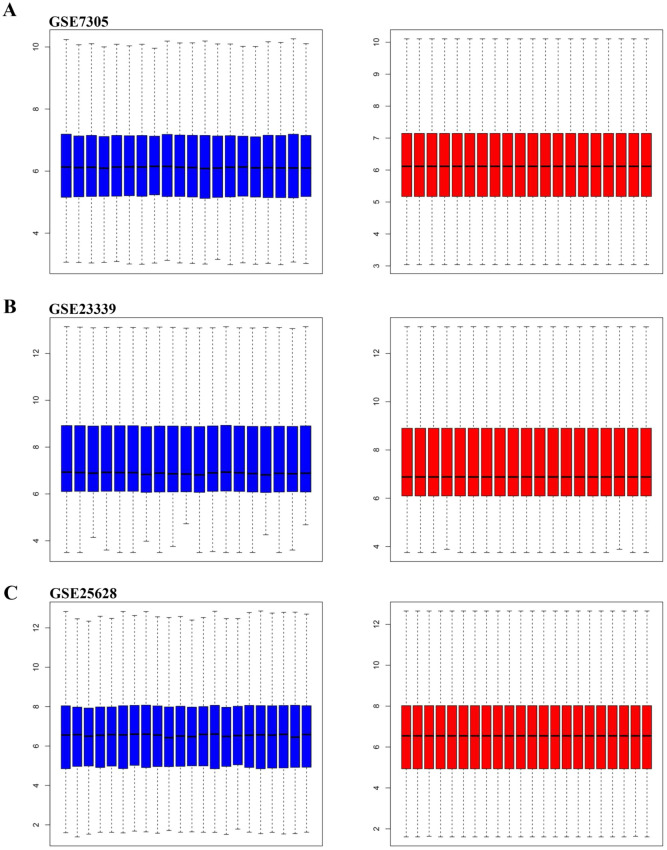
Box plot graph of value distribution of GSE7305 (**A**), GSE2339 (**B**) and GSE25628 (**C**) The left side is before normalization, the right side is after normalization.

**Figure 2:**
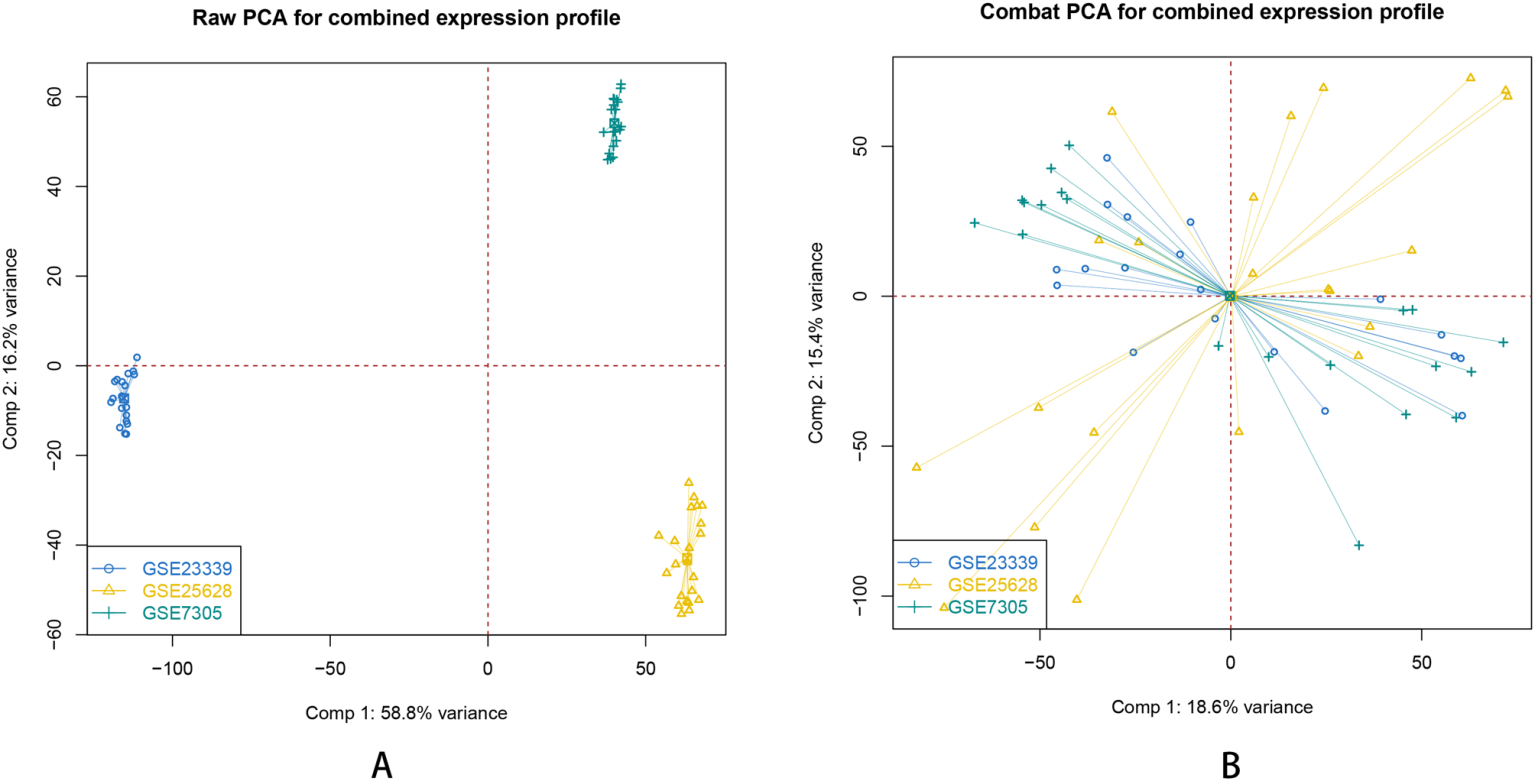
Principal Component Analysis for combined expression profile of each data set. (**A**) raw PCA for combined expression profile before eliminating the inter-batch difference. (**B**) combat PCA for combined expression profile after eliminating the inter-batch difference.

**Figure 3:**
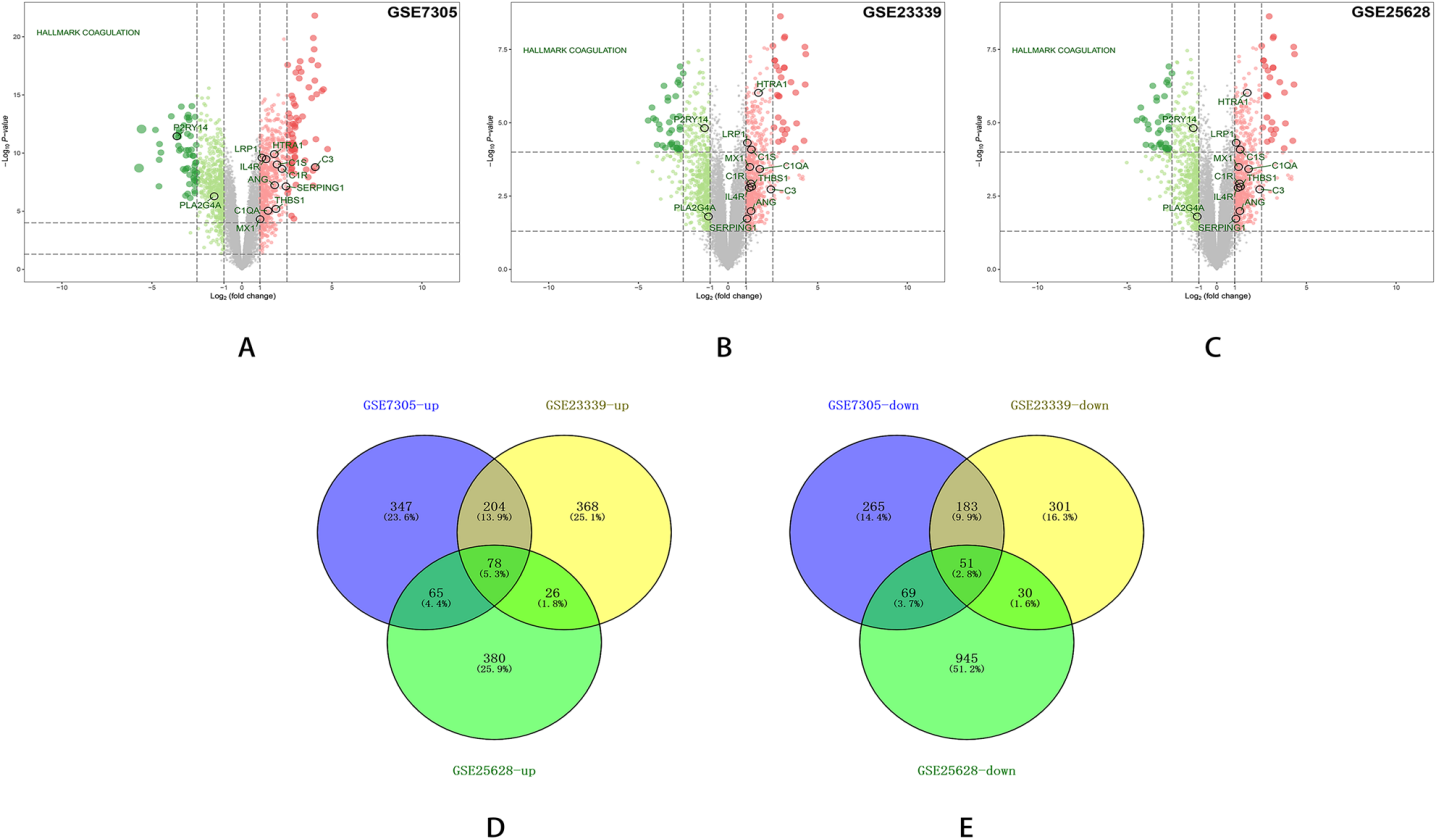
Volcano plots and Venn diagrams of DEGs in endometriosis microarray datasets. Volcano plots showing DEGs in GSE7305 (**A**), GSE23339 (**B**), and GSE25628 (**C**). DEGs are those genes with P-value < 0.05 and |log (FC)|> 1. Red indicates relative up-regulated genes and green indicates down-regulated genes. Venn diagrams of up-regulated (**D**) or down-regulated (**E**) DEGs from these three datasets, as indicated.

**Figure 4:**
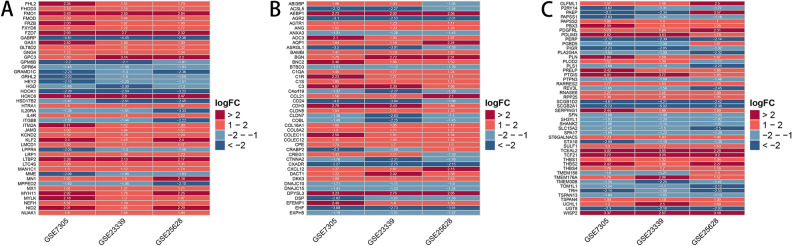
Heat maps of the DEGs in endometriosis microarray datasets. The vertical column is the name of the data set, the horizontal column is the gene name. DEGs are those genes with P value < 0.05 and |log (FC)|> 1. Red indicates up-regulation and blue indicates down-regulation.

Samples in each dataset (GSE7305, GSE23339, and GSE25628) were normalized using Robust Multi-array Average (RMA) function in the Affy package (Fig. 1). All datasets were normalized using SVA package to eliminate potential inter-batch difference (Fig. 2). Then we used the Limma package to perform differential expression analysis on the three data sets with a definition criterion based on p < 0.05 and |log (FC)|> 1 to select statically statistically DEGs. In GSE7305, 1262 DEGs were identified, of which 694DEGs were up-regulated and 568 down-regulated (Fig. 3A); In GSE23339,1241 DEGs were identified, of which 676DEGs were up-regulated and 565 down-regulated (Fig. 3B); In GSE25628, 1644 DEGs were identified, of which 549DEGs were up-regulated and 1095 down-regulated (Fig. 3C). We identified 129 DEGs, of which 78 were up-regulated and 51 down-regulated (Fig. 3D,E and Table 2). Then, we converted the chart into the heat map to show the expression of DEGs intuitively (Fig. 4).

**Table 2:** 129 DEGs in EMs were identified by integrated bioinformatics, with 78 up-regulated and 51 down-regulated.

DEGs	Gene names
Up-regulated	CXCL12/OLFML1/MAN1C1/FHOD3/BAMBI/HTRA1/PDGFRL/RARRES2/AEBP1/AM3/BGN/DKK3/NID2/FMOD/LTBP2/LRP1/DACT1/MX1/FMO1/FXYD6/MYLK/MN1/GLT8D2/CCL21/WISP2/C1S/DPYSL3/KLF2/KCND2/C1QA/PBX3/C1R/THBS1/PTGIS/FHL2/THBS2/ITM2A/IL4R/NUAK1/RNASE6/AQP1/PRELP/COLEC12/TCF21/TSPAN4/GNG4/UCHL1/FRZB/FZD7/LTC4S/TMEM176A/HOXC6/GAS1/CLDN5/SULF1/TCEAL2/MYH11/COL16A1/COL8A2/SERPING1/PAPSS2/CPE/BNC2/CDH3/RPP25/NEFH/THBS4/GPC3/AGTR1/AOC3/EFEMP1/LMCD1/PDLIM3/ANG/ST6GALNAC5/C3/COLEC11/PLN
Down-regulated	EHF/HOOK1/EXPH5/ITGB8/IL20RA/PLS1/REV3L/TOM1L1/CXADR/UGT8/SH3YL1/PLA2G4A/BTBD3/GRAMD1C/MPPED2/PERP/ANXA3/DNAJC15/SLC15A2/DNAJC10/SPA17/PTPN3/DSP/TRH/PIGR/GPR64/GRHL2/TMEM30B/TSPAN13/HEY2/SHANK2/STX18/PAPSS1/GABRP/MME/HSD17B2/CLDN7/ACSL5/COBL/CD24/SFN/P2RY14/SCGB2A1/AGR2/ASRGL1/C4orf19/LPPR4/SCGB1D2/GPM6B/CTNNA2/HGD

### Protein–protein interaction (PPI) network analysis in DEGs

**Figure 5:**
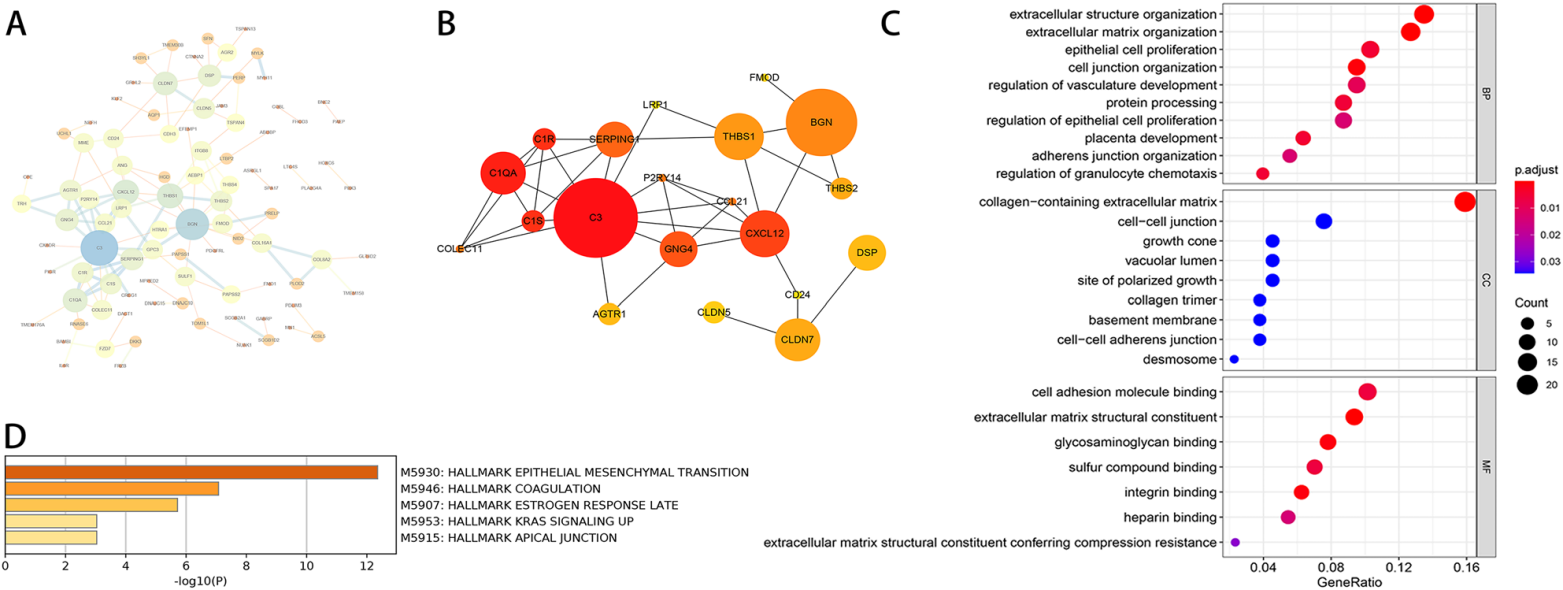
(**A**) PPI network analysis of DEGs. (**B**) PPI network analysis of the top 20 hub genes with the highest scores. The size of the circle is related to the score. (**C**) GO analysis of DEGs in endometriosis. The result of GO analysis was divided into three parts: biological process (BP), cellular component (CC), and molecular functions (MF). The color of the circle was related to the P-value and the size of the circle was related to the count. (**D**) Hallmark pathway enrichment of DEGs in endometriosis. Pathway enrichment of DEGs in endometriosis was visualized on a bar chart, showing -Log10 (P-value).

PPI network was performed using the online STRING database and then Cytoscape software was used for visualization. After removing partly connected and isolated nodes, we constructed a grid network using the Cytoscape-software (Fig. 5A). Then, we used the Cytohubba module of Cytoscape to calculate the top 20 hub genes with the highest scores, including SERPING1, P2RY14, C1S, C3, C1QA, C1R, GNG4, CCL21, CXCL12, COLEC11, AGTR1, LRP1, THBS1, BGN, FMOD, THBS2, DSP, CD24, CLDN5, CLDN7 (Fig. 5B).

### Gene Ontology (GO) functional enrichment in DEGs

Thereafter, we performed gene ontology (GO) enrichment analysis of DEGs in endometriosis by using clusterprofiler package. The results were divided into three parts: biological process (BP), cellular component (CC), and molecular functions (MF). Then, we use R to import the top ten results of every sector into the figure (Fig. 5C). In the BP, DEGs were mainly involved in cell adhesion, epithelial cell proliferation, granulocyte chemotaxis regulation, and extracellular matrix remodeling. In the CC, cell connection, cell adhesion, and extracellular matrix collagen-containing were included. In the MF, terms were mainly involved in binding and extracellular matrix structural constituent conferring compression resistance.

### Hallmark signaling pathway enrichment in DEGs

We use Metascape16 to perform signaling pathway enrichment of DEGs in endometriosis, and then we submit the most significantly enriched pathways to Hallmark genes hit analysis. Through Hallmark pathway enrichment analysis, epithelial-mesenchymal transition (EMT), coagulation, estrogen response late, KRAS signaling up, and apical junction were identified (Fig. 5D). Among them, EMT and coagulation pathways showed great significance according to the P Value (Table 3).

**Table 3:** Hallmark pathway enrichment analysis of DEGs.

Term	Description	counts	Log(P)	genes
M5930	Epithelial mesenchymal transition	15	−12.356	BGN, ABI3BP, DPYSL3, HTRA1, GAS1, MYLK, LRP1, FMOD, THBS1, NID2, THBS2, COL8A2, COL16A1, CXCL12, PLOD2
M5946	Coagulation	13	−7.076	SERPING1, P2RY14, C1S, C3, C1QA, C1R, MX1, IL4R, ANG, LRP1, THBS1, HTRA1
M5907	Estrogen response late	12	−5.716	PAPSS2, SFN, PDLIM3, CCN5, CXCL12, TSPAN13, PERP, CPE, AGR2, CLDN7, FHL2, MPPED2
M5953	Kras signaling up	6	−3.036	BTBD3, TMEM158, TSPAN13, CPE, TMEM176A, PIGR
M5915	Apical junction	6	−3.036	CDH3, JAM3, CLDN7, COL16A1, CLDN5, TSPAN4

### Bioinformatics analysis shows that SERPING1, C1S, C1QA, C1R, C3, and TF are up-regulated in EMs while P2RY14 is down-regulated in EMs group

Combined Top20 hub genes with pathway enrichment analysis, coagulation pathway showed great significance. Meanwhile, SERPING1, P2RY14, C1S, C1QA, C1R, and C3 showed better coagulation pathway correlation and EMs correlation. Among them, SERPING1, C1S, C1QA, C1R, and C3 were up-regulated in EMs while P2RY14 was down-regulated (Figure S2a). To verify whether the expression of genes involved in the coagulation cascade was synchronized with complement factors, we selected tissue factor (TF) for verification, which was the important factor in the exogenous coagulation pathway and EMs^25,26^. After analyzing the data set, TF was found to be up-regulated in EMs (P = 0.044) (Figure S2b).

### The expression of SERPING1, C1S, C3, C1QA, C1R, and TF are up-regulated while P2RY14 is down-regulated at mRNA level and protein level in EMs group

**Figure 6:**
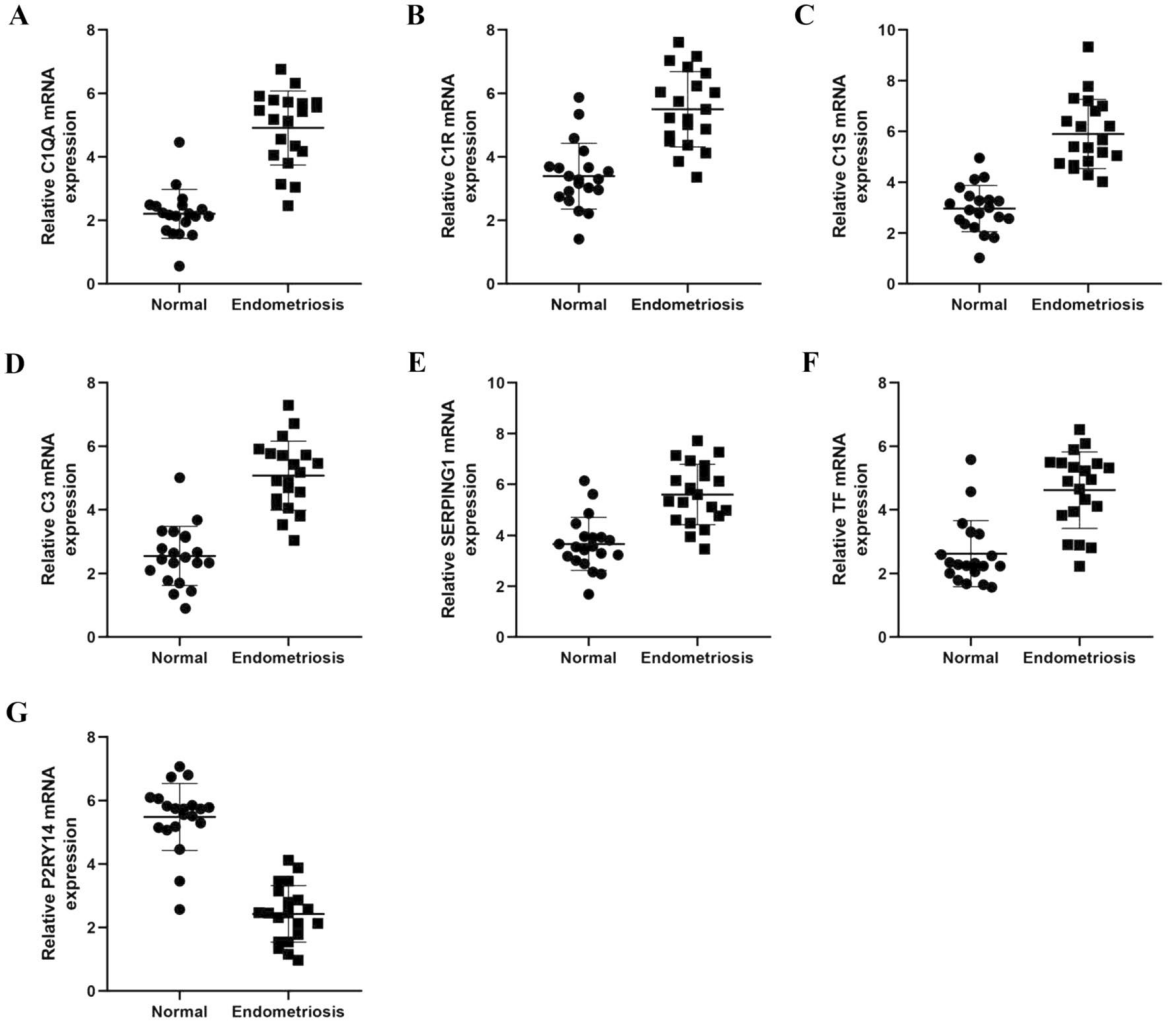
The mRNA expression of hub genes between groups by qPCR. (**A**–**E**) the expression of SERPING1, C1S, C1QA, C1R, and C3 were frequently up-regulated in the EMs group compared with the normal group. (**F**) the expression of TF was up-regulated in EMs group compared with the normal group. (**G**) the expression of P2RY14 was down-regulated in EMs group compared with the normal group.

**Figure 7:**
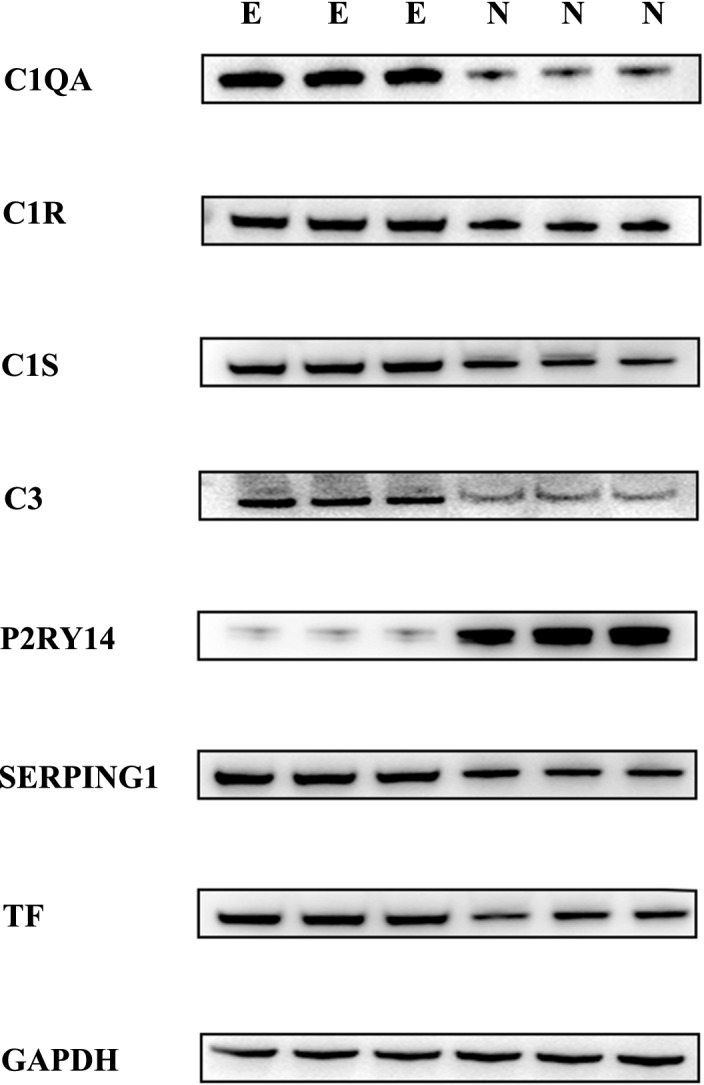
The protein expression of hub genes between groups by WB. The expression of SERPING1, C1S, C1QA, C1R, C3, and TF were up-regulated in EMs group compared with the normal group. The expression of P2RY14 was down-regulated in EMs group compared with the normal group.

**Figure 8:**
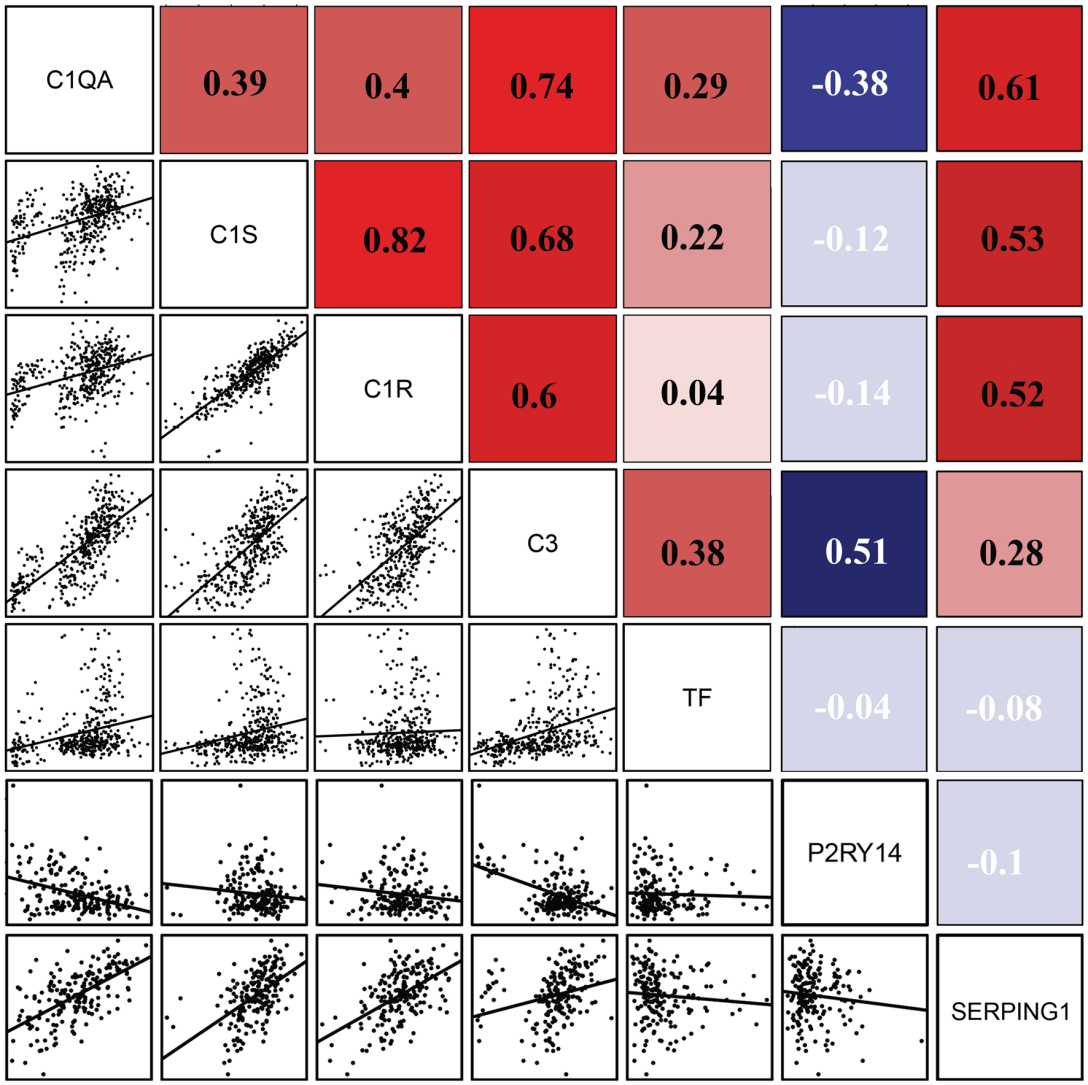
Gene co-expression correlation analysis. The expression of central complement factors was positively related to TF while SERPING1 and P2RY14 were negatively related to TF. Red represents a positive correlation and blue indicates a negative correlation.

We used qPCR to analyze the mRNA expression level of the hub genes in the coagulation pathway. The expression of SERPING1, C1S, C1QA, C1R, and C3 were up-regulated in EMs group (Fig. 6A–E) while P2RY14 was down-regulated (Fig. 6G). This result was also validated by western blotting at the protein level (Fig. 7). The verification of the hub genes both at mRNA level and protein level suggested the abnormal status of complement in EMs. We also found that the expressions of TF were up-regulated both at the mRNA and protein levels of EMs group (Fig. 6F, 8).

### Bioinformatics analysis shows that Central complement factors (C1S, C1QA, C1R, C3) are positively related to TF

Further, we analyzed the correlation between the expression of hub genes and TF in GSE141549 data set. In co-expression correlation analysis, the complement showed a positive correlation with TF while SERPING1 and P2RY14 showed a negative correlation with TF. Interestingly, SERPING1 was up-regulated in EMs synchronized with complement components (Fig. 8). Also, these genes were involved in the coagulation, proposing the existence of crosstalk between complement and coagulation cascades in EMs.

## Discussion

Here, through integration and analysis of the data from 3 microarray datasets, we identified 129 DEGs, of which 78 up-regulated and 51 down-regulated in EMs. GO functional enrichment analysis showed that DEGs are mainly enriched in cell adhesion, extracellular matrix remodeling, chemokine regulation, angiogenesis regulation, epithelial cell proliferation, which have been proved the essential steps in the development of EMs^26–29^. In this study, Hallmark pathway enrichment analysis disclosed coagulation and epithelial-mesenchymal transition (EMT) were the most significant pathways in EMs^30–32^. To EMT, many studies have revealed its main role in the development of EMS and Yihua Wang et al. have also confirmed this by bioinformatic analysis^33^.

So, in this study, we focused on the coagulation pathway of EMS and found that complement factors were enriched in this way according Hallmark pathway enrichment analysis. It is widely known that the relationship between the immune system and EMS is intimate^34,35^. Swati et al. revealed that chronic inflammation in endometriosis is dominated by complement^7^. Sikora et al. had proved that abnormal activation of the complement pathway occurred in EMS and resulted in the overexpression of C1Q, MBL, C1INH^36^. In our study, we found that the up-regulated expression of the central complement factor (C1S, C1QA, C1R, and C3) in EMS, indicating that the abnormal status of complement might be involved in the pathogenesis of EMS. Meanwhile, Vigano et al. confirmed the existence of hypercoagulable state in EMS patients^37^. Researches conducted by Guo et al. also revealed that stromal cells in EMS lesions could secrete thrombin and Thromboxane A2 (TXA2) to induce platelet activation and participate in coagulation^38,39^. And then, Thrombin in the endometrial matrix could be activated in response to repeated bleeding from highly vascularized lesions in EMs and combined with PAR-1 to mediate thrombin-induced angiogenesis^40,41^. By now, few researches focused on EMs indicated the existence of the crosstalk between complement and coagulation.

Though, the crosstalk between the complement and coagulation cascades has been proved in septicemia. Rittirsch revealed that complement activation could enhance coagulation through up-regulating the expression of tissue factor^42^. Hence, we proposed that TF might be the interaction between complement and coagulation and the crosstalk between complement and coagulation might also play an essential role in the development of EMS. Previously, Ding et al. found that the concentration of TF in the peritoneal fluid of EMS patients is higher than that of normal women^43^. TF had also been proved to be overexpressed in ectopic lesions^44^. Lin et al. found that TF was overexpressed in epithelial cells and stromal cells of EMs^45^. Graciela et al. found that markedly increased expression of TF in glandular epithelial cells of ectopic and eutopic endometrium derived from patients with EMS^25^. It is generally known that TF could combine with circulating factor VII-a to participate in the exogenous coagulation pathway. Therefore, we considered that TF might be a bridge link between complement and coagulation in EMS. Two mechanisms might account for such an association. Firstly, when membrane attack complex (MAC) was anchored on the cell surface, MAC could stimulate oxidation of protein disulfide isomerase and induce exposure of functionally active procoagulant TF^46^. Secondly, MAC could enhance the platelet procoagulant reaction by generating procoagulant particles or inverting the membrane to expose phosphatidylserine^47^. Importantly, in addition to participating in blood clotting, activated platelets could be recruited to lesions and derive TGF- β to promote the development of diseases^32,48^.

Moreover, TF could induce the formation of blood vessels through AKT-ETS1 and promotes the stability of blood vessels through PAR2-SMAD3, which might be beneficial to the survival of lesions^49,50^. Nowadays, TF targeted treatments have shown a good therapeutic effect, which could significantly inhibit the EMS lesion^51–53^. In our experimental verification, TF was also found to be overexpressed in EMS. Combined with co-expression analysis, we demonstrated that the up-regulated expression of complement factors was positively related to TF. The result indicated the existence of crosstalk between the complement and coagulation cascade. So far, however, there is still little evidence focused on the interaction of complement and coagulation cascade in EMS. Subsequently, the encouraging result discovered by our study may provide a novel direction for future researches.

Besides, SERPING1 and P2RY14 were also genes of great significance in our analysis. SERPING1 is the coding gene of C1NH, which is the inhibitor of the complement pathway^54,55^. And, SERPING1 was found to be overexpressed in EMS synchronized with C1S, C1QA, C1R, and C3, which showed a dual tendency to pro-inflammatory and anti-inflammatory. It is widely considered that EMS is a chronic inflammatory disease while Zhou et al. proposed that anti-inflammatory was equally important in EMS and that Anti-inflammatory factors involved in EMS could promote the growth, invasion, angiogenesis and immune escape of lesions^56^. This phenomenon has also been reported at the cell level. In general, the M2 macrophage phenotype accounts for anti-inflammation, whereas the M1 macrophage phenotype is proinflammatory. Interestingly, the ratio of M2 increased as EMS progressed^57^. Up-regulated expression of SERPING1 suggested that there may be protective mechanisms for ectopic lesions in vivo. P2RY14 is a membrane receptor for UDP-glucose, which is exclusively expressed in the epithelium. It could induce innate mucosal immunity in the female reproductive tract independent of the pathogen recognition pattern^58^. In our research, P2RY14 was down-regulated, which might be related to immune surveillance escape.

Through bioinformatics analysis, our research proposed that TF might be a significant bridge link between complement and coagulation in EMS. Meanwhile, we proved the abnormal state of complement in EMS again. To the best of our knowledge, this is an important study concentrating on the interaction of TF and the central complement factor in EMS. However, our study inevitably had certain limitations. Firstly, the amount of data published in GEO was limited, which might lead to potential errors or bias. In subsequent studies, the samples should be further expanded to reduce bias. Secondly, there were regional differences in data sources, so the research results might not apply to all patients. Hence, an in-depth research is needed to be verified.

## Conclusion

Taken as a whole, through serial bioinformatics analysis and verification experiments, we confirmed the abnormal status of complement in EMs. And then, SERPING1, P2RY14, C1S, C1QA, C1R, and C3 might become potential immunotherapy targets for EMs. Besides, TF connected the central complement factors and coagulation cascades and was confirmed to be positively related to complement. Hence, the crosstalk between the complement and coagulation might play an essential role in the development of EMs.

## Supplementary Information

Supplementary Information 1.

Supplementary Information 2.
