# Supplementary material for: Multi‐omics analysis reveals the interaction between the complement system and the coagulation cascade in the development of endometriosis
Source: Sci Rep. 2021 Jun 7;11:11926. doi: 10.1038/s41598-021-90112-x (PMC8185094; doi:10.1038/s41598-021-90112-x)

Figure S1. Gene co-expression correlation analysis in raw data set.

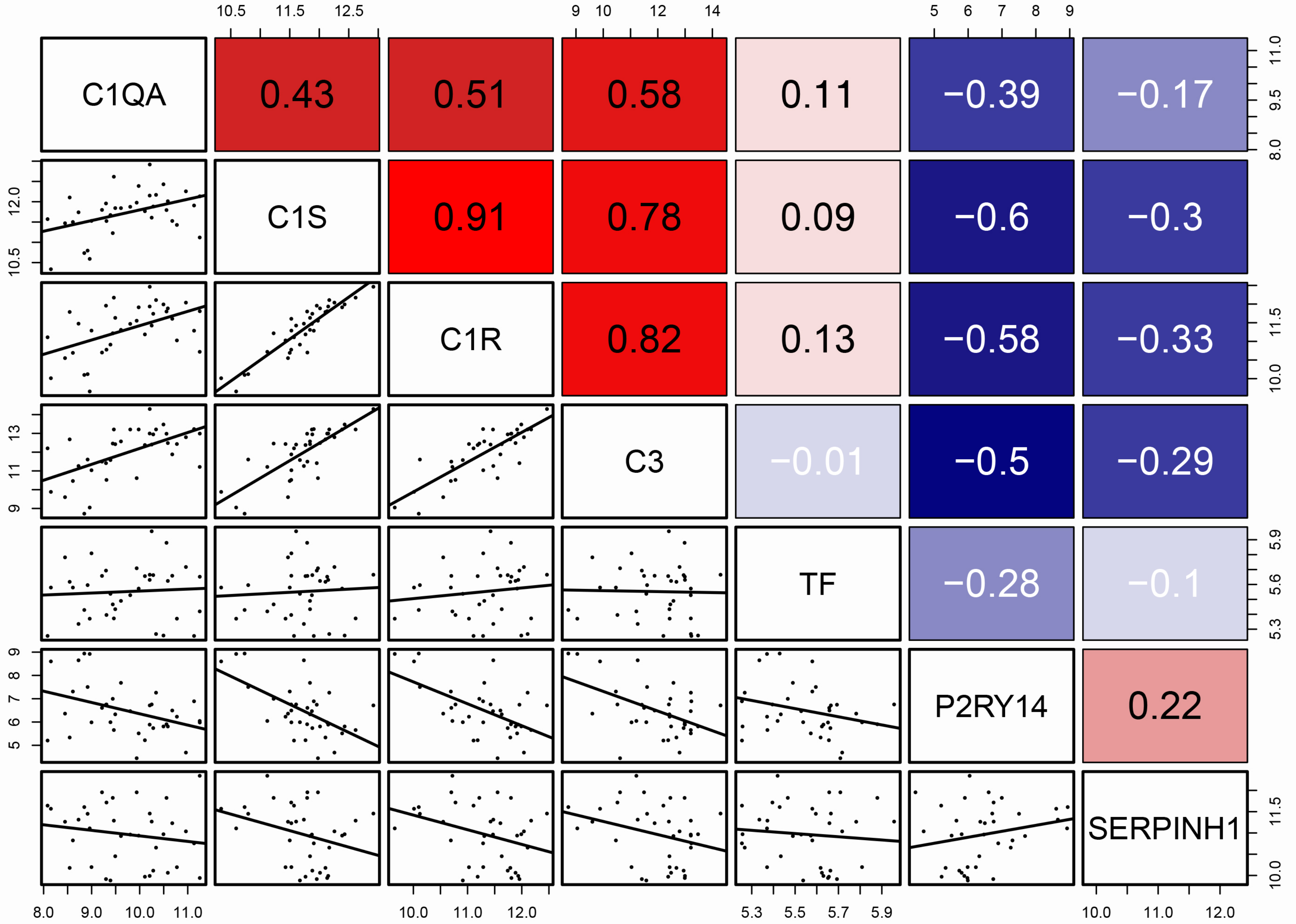

Figure S2. Bioinformatics analysis of the hub's expression between groups

A

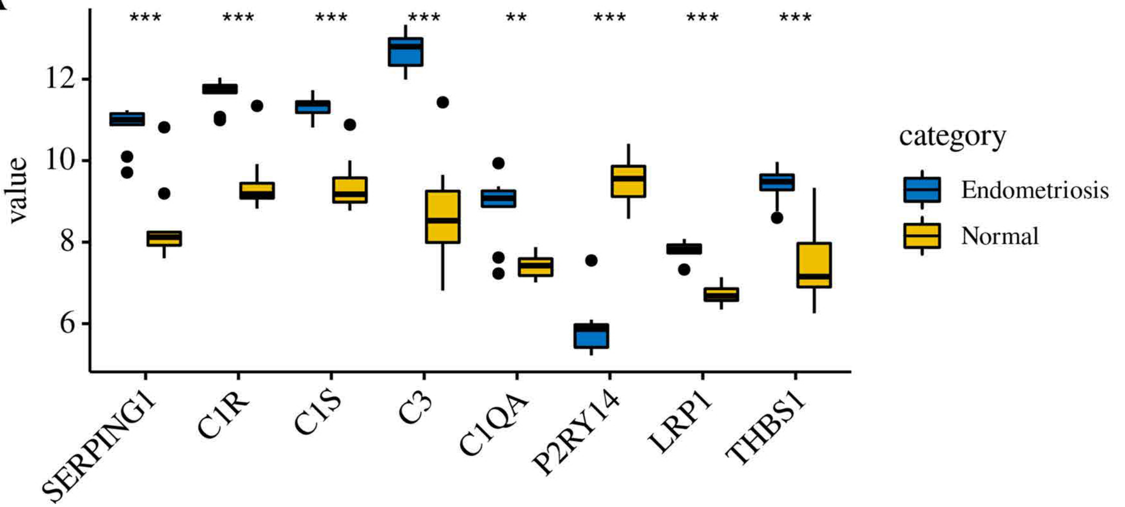

B

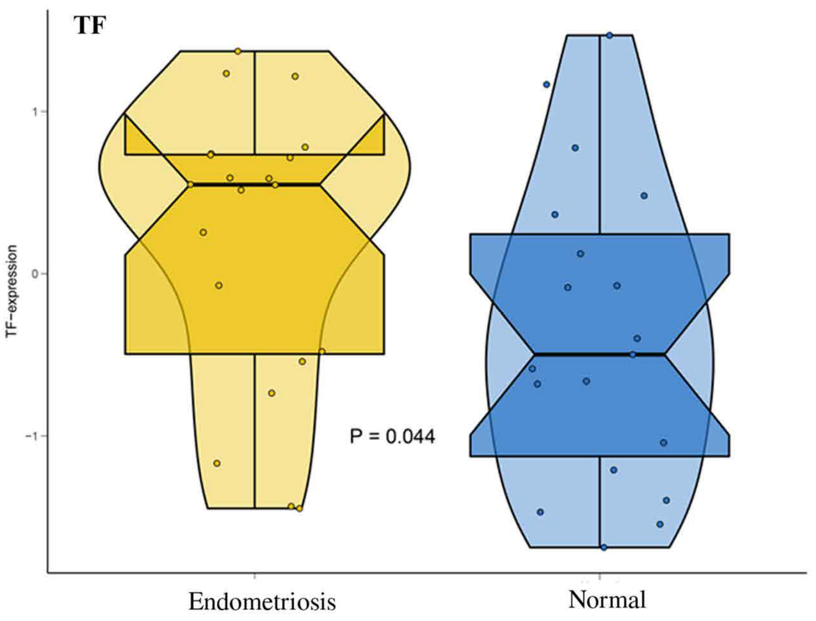

Supplement: Supplementary file 1 — Supplementary Information 1. [file 41598_2021_90112_MOESM1_ESM.pdf]
