# Supplementary figures and images for: Multi‐omics analysis reveals the interaction between the complement system and the coagulation cascade in the development of endometriosis
Source: Sci Rep. 2021 Jun 7;11:11926. doi: 10.1038/s41598-021-90112-x (PMC8185094; doi:10.1038/s41598-021-90112-x)

C1QA

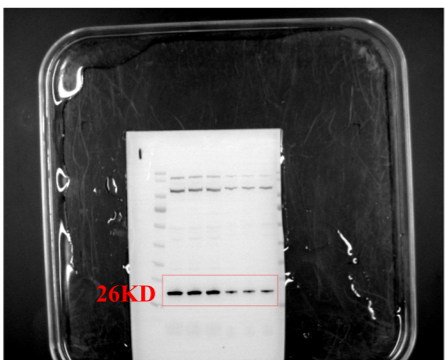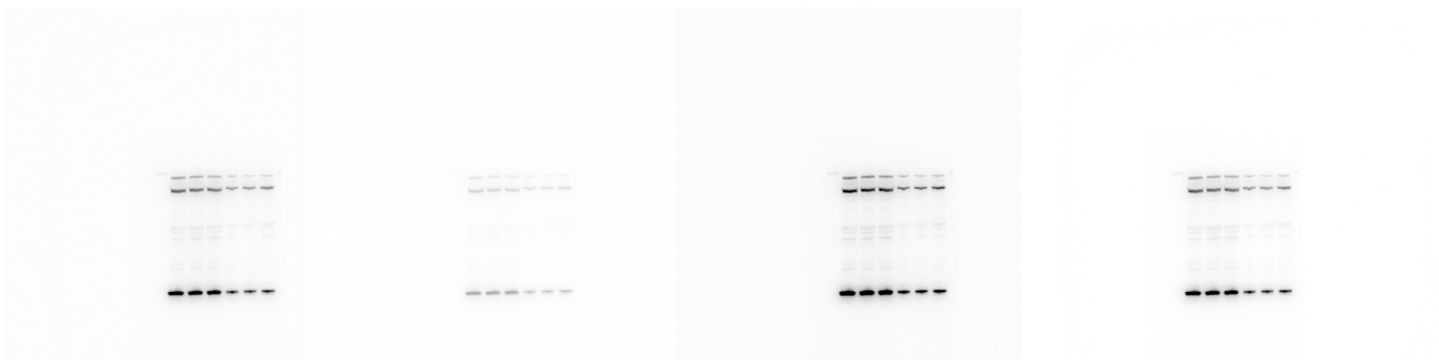

C1R

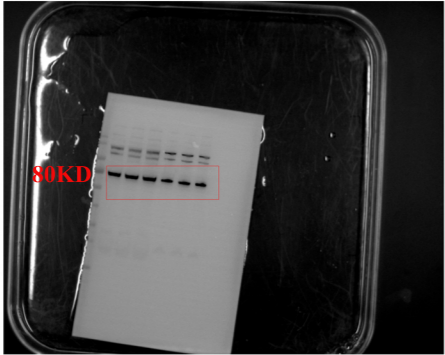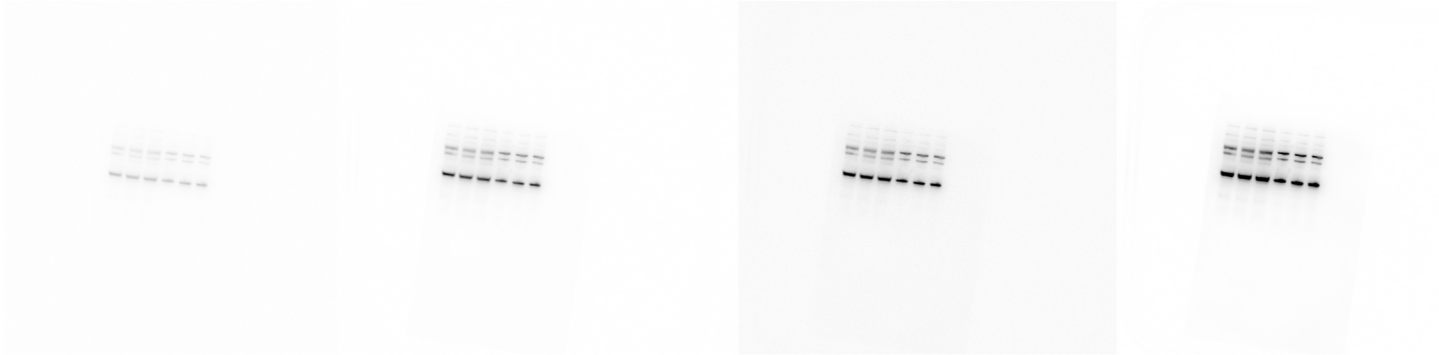

C1S

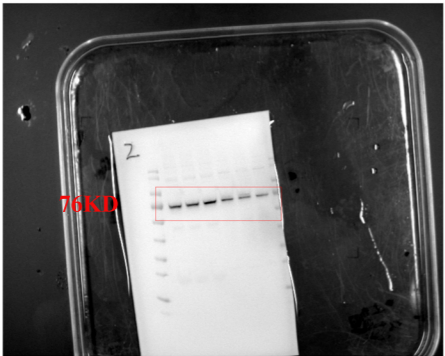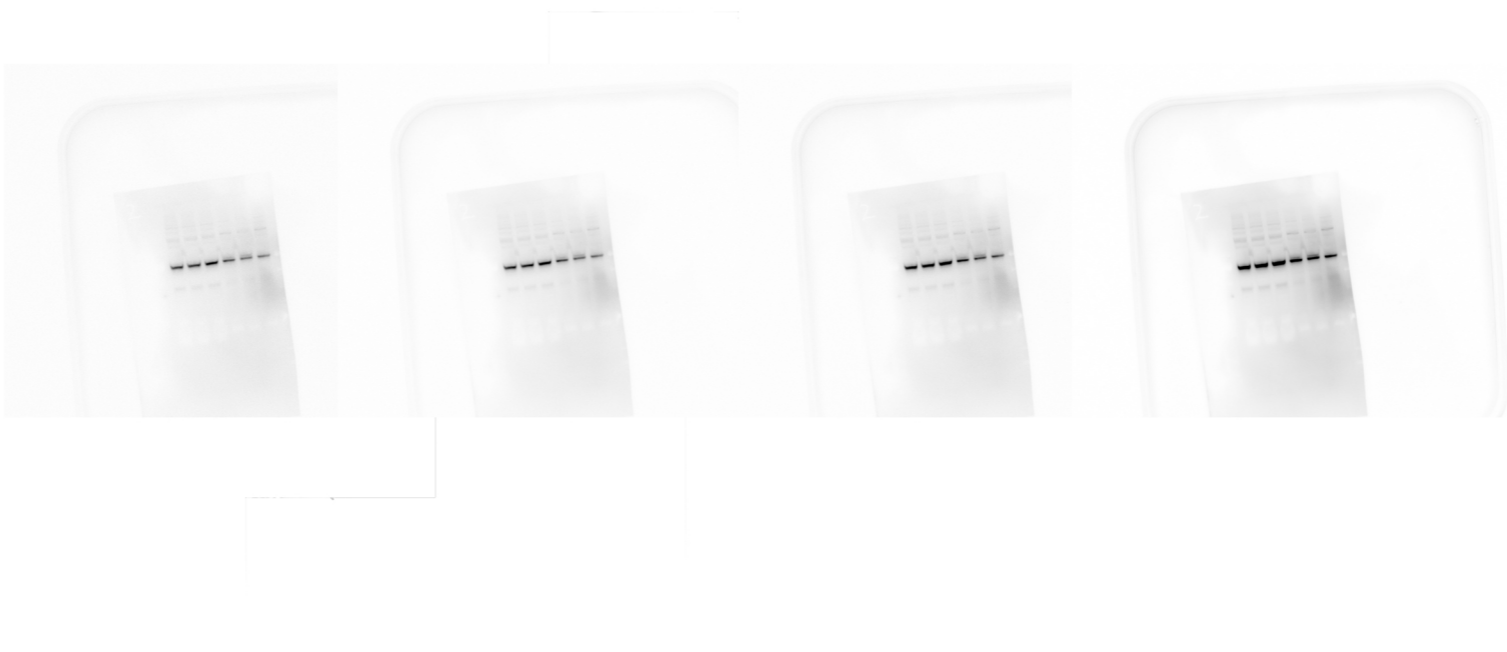

C3

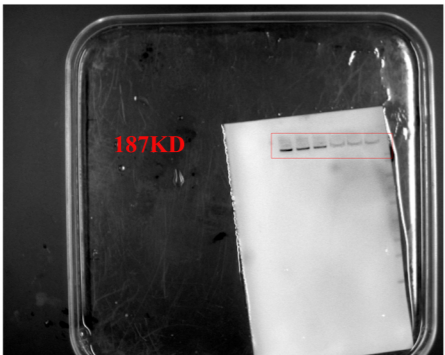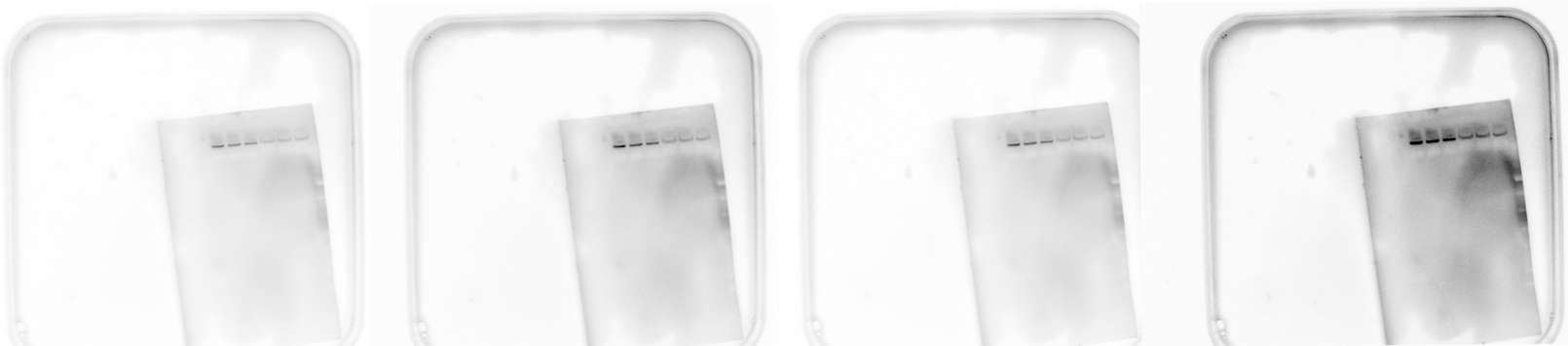

P2RY14

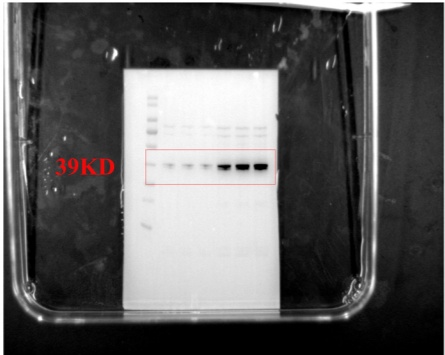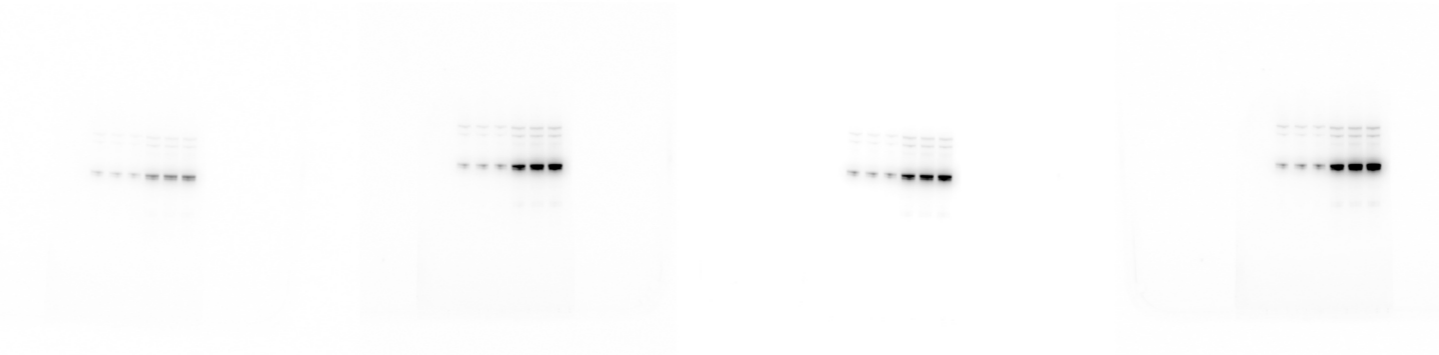

SERPING1

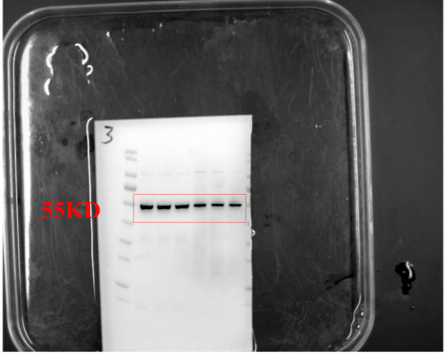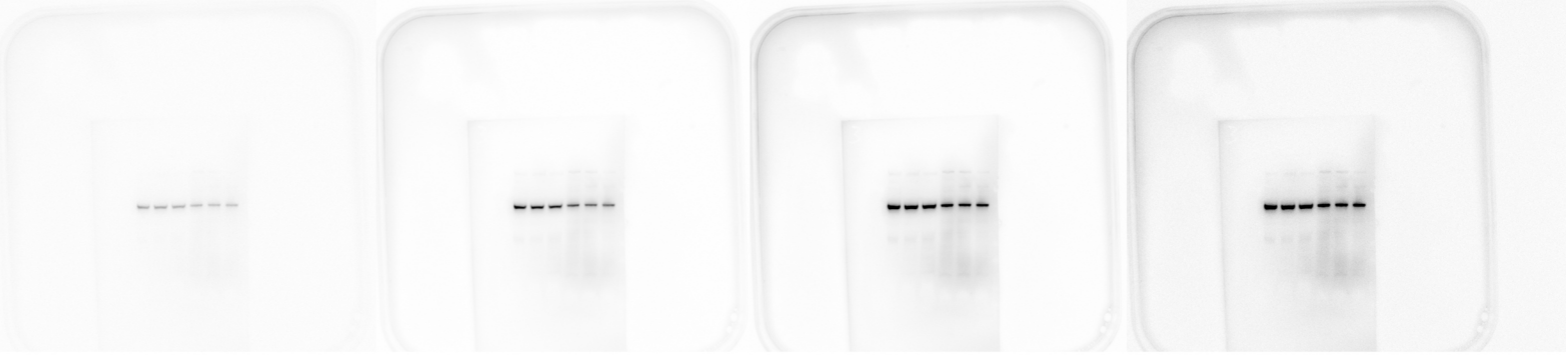

TF

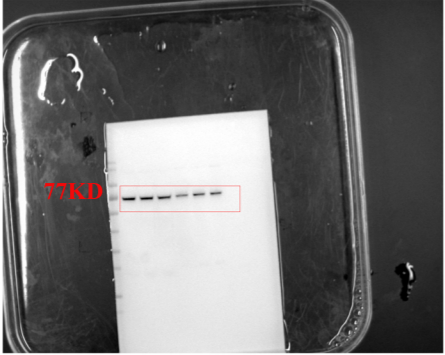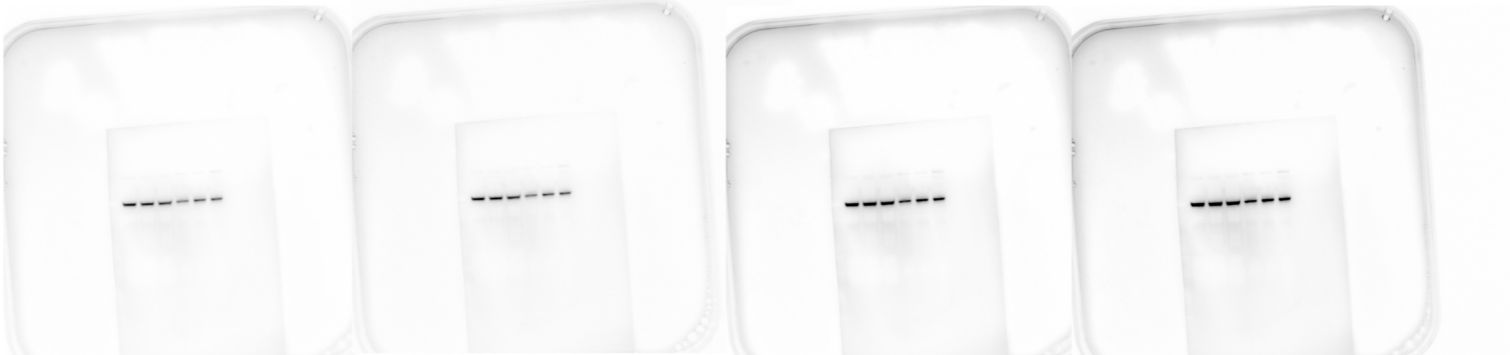

GAPDH

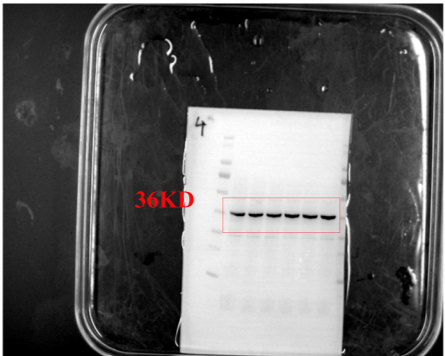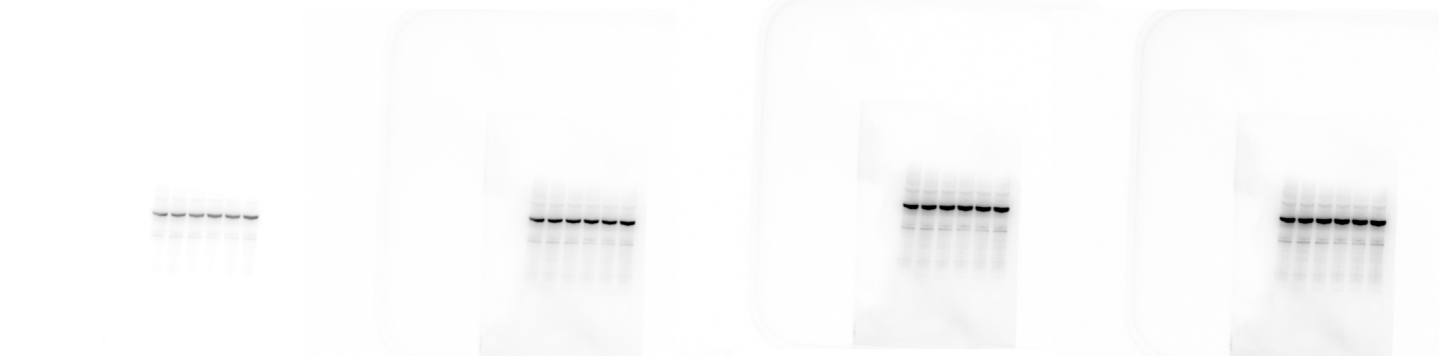

Supplement: Supplementary file 2 — Supplementary Information 2. [file 41598_2021_90112_MOESM2_ESM.pdf]
